# A novel nonsense mutation in *MYO15A* is associated with non-syndromic hearing loss: a case report

**DOI:** 10.1186/s12881-018-0657-y

**Published:** 2018-08-01

**Authors:** Di Ma, Shanshan Shen, Hui Gao, Hui Guo, Yumei Lin, Yuhua Hu, Ruanzhang Zhang, Shayan Wang

**Affiliations:** Shenzhen People’s Hospital, Clinical Medical College of Jinan University, Dongmen North Rd. 1017, Shenzhen, 518020 People’s Republic of China

**Keywords:** Hearing loss, DFNB3, *MYO15A*, Nonsense mutation

## Abstract

**Background:**

Hearing loss is genetically heterogeneous and is one of the most common human defects. Here we screened the underlying mutations that caused autosomal recessive non-syndromic hearing loss in a Chinese family.

**Case presentation:**

The proband with profound hearing loss had received audiometric assessments. We performed target region capture and next generation sequencing of 127 known deafness-related genes because the individual tested negative for hotspot variants in the *GJB2*, *GJB3*, *SLC26A4*, and *MTRNR1* genes. We identified a novel c.6892C > T (p.R2298*) nonsense mutation and a c.10251_10253delCTT (p.F3420del) deletion in *MYO15A*. Sanger sequencing confirmed that both mutations were co-segregated with hearing loss in this family and were absent in 200 ethnically matched controls. Bioinformatics analysis and protein modeling indicated the deleterious effects of both mutations. The p.R2298* mutation leads to a truncated protein and a loss of the functional domains.

**Conclusions:**

Our results demonstrated that the hearing loss in this case was caused by novel, compound heterozygous mutations in *MYO15A*. The p.R2298* mutation in *MYO15A* was reported for the first time, which has implications for genetic counseling and provides insight into the functional roles of *MYO15A* mutations.

**Electronic supplementary material:**

The online version of this article (10.1186/s12881-018-0657-y) contains supplementary material, which is available to authorized users.

## Background

Hearing loss is one of the most prevalent neurosensory defects in the general population. On average, about 1–2 in 1000 newborns have congenital or prelingual deafness [1, 2]. Among the common causes of deafness, approximately 50–60% can be attributed to genetic factors [3]. Hereditary deafness can be classified as being syndromic (about 30%) or non-syndromic (about 70%) [4]. Autosomal recessive inheritance states account for 77–93% of hereditary hearing loss [5]. To date, 105 loci associated with autosomal recessive non-syndromic hearing loss (DFNB) have been mapped and 67 genes have been identified (http://hereditaryhearingloss.org). In particular, *GJB2*, *SLC26A4*, *MYO15A*, *OTOF*, and *CDH23* are the most common genes responsible for hereditary hearing loss [5, 6].

Mutations in *MYO15A* can lead to DFNB3 (OMIM: 600316). By performing a genome-wide disequilibrium study in 1995, *MYO15A* was found to be associated with deafness for the first time in Indonesian residents [7, 8]. *MY015A* encodes an unconventional MYO15A protein, which is a structural constituent of hair cells and stereocilia. Clinically, mutations in *MY015A* have manifested as profound, congenital, non-syndromic hearing loss [9]. With the extensive application of next generation sequencing, genetic diagnosis is playing a more important role in the prenatal diagnosis and clinical evaluation of *MYO15A*-associated hereditary hearing loss [10, 11]. However, there are still a large number of variants of undetermined significance, which makes the interpretation of variant effects challenging.

In the present case, we collected and analyzed the clinical genetics data from a family with autosomal recessive non-syndromic hearing loss (ARNSHL). We identified compound heterozygous mutations in *MYO15A* including a novel nonsense mutation, which induced pathogenic effects on the protein function and therefore should be responsible for the deafness in this family.

## Case presentation

### Clinical manifestations

**Fig. 1:**
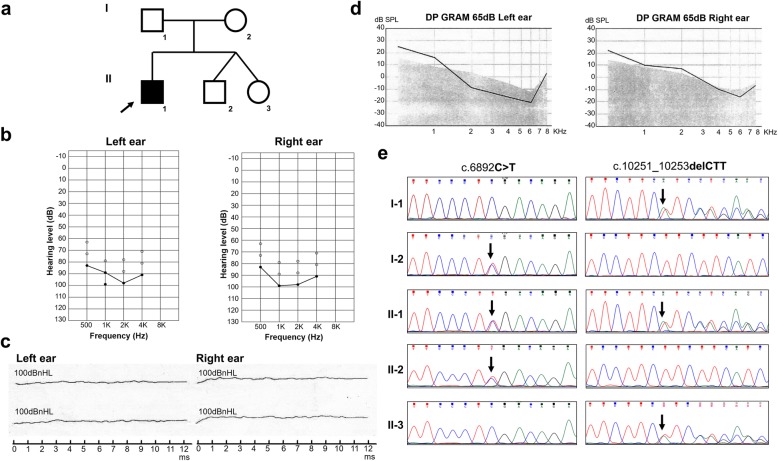
Molecular genetics and clinical analysis. **a** Family pedigree diagram. Filled symbols and opened symbols indicate affected and unaffected individuals, respectively. Arrows indicate the proband in this family. **b** Auditory steady state response (ASSR) audiogram of left and right ears of the affected proband showed profound hearing loss. **c** Auditory brainstem response (ABR) demonstrated no waveforms response for click stimulus at 100 dB HL of both ears of the proband. **d** Distortion product otoacoustic emission (DPOAE) audiogram of both ears of the proband. The DPOAE were detected in 500, 1,000, 2,000, 4,000, 60,00, and 8000 Hz frequencies. The stimulus tolerance is 5 dB above noise level. **e** Sanger sequencing results of the c.6892C > T and c.10251_10253delCTT variants in all family members. Arrows indicate the position of the nucleotide changes identified in this study. I-2, II-1, II-2 carried the 6892C > T mutation in *MYO15A*, and I-1, II-1, II-3 carried the c.10251_10253delCTT mutation in *MYO15A*

A Chinese family with ARNSHL was recruited from the Shenzhen People’s Hospital Prenatal Diagnosis Center. Five family members including three males and two females, which spanned two generations, participated in this study. Clinical and audiometric assessments were performed by an experienced otolaryngologist. The proband (II-1, Fig. 1a) with congenital bilateral profound deafness is a 6-year-old boy. He first presented no response to sound when he was 3 months old. He failed the auditory brainstem responses (ABR), auditory steady state response (ASSR) and distortion product otoacoustic emission (DPOAE) tests (Fig. 1b-d). His ASSR audiogram results showed severe hearing loss in both ears. Moreover, even with stimulation at 100 dB, no apparent waveforms were aroused when performing the ABR test. Temporal bone computed tomography (CT) scan and magnetic resonance imaging (MRI) were both performed to exclude other inner ear malformations. His parents (I-1 and I-2, Fig. 1a), who underwent pure-tone audiometry, showed normal hearing. There was no family history of congenital hearing loss recorded and putative environmental factors could be excluded. At the age of 1 year and 9 months, the patient became significantly delayed in speech and developed prelingual ARNSHL, and therefore received a cochlear implantation. After that, he obtained a speech rehabilitation training for 1 year. At the time of re-examination, the proband was 4 years old and his mother was pregnant with dizygotic twins. Informed consent was obtained from the mother to collect the amniotic fluid for both dizygotic twins. Peripheral blood samples were collected from the proband and his parents. At present, the auditory performance and speech intelligibility of the proband have greatly improved. Moreover, the dizygotic twins are 2 years old now and both of them passed the newborn hearing screening tests suggesting normal hearing.

### Identification of *MYO15A* mutations

Reverse dot blotting for 16 hotspot variants in the *GJB2*, *GJB3*, *SLC26A4*, and *MTRNR1* genes was performed to rule out common variants in hereditary hearing loss. Next, target genomic capturing and next-generation sequencing for a panel of 127 genes related to hereditary hearing loss, which included the *GJB2*, *GJB6*, *SLC26A*, *MTRNR1*, and *MTTS1* genes, were conducted to screen for the causative gene variant (s) of deafness in this family (see Additional file 1: Table S1 and Additional file 2: Table S2). DNA from the proband was extracted and analyzed by the illumina Hiseq2000 platform [12] (BGI, Shenzhen, China). Reads were aligned to the UCSC (http://www.genome.ucsc.edu) hg19 human reference genome using BWA software. GATK software was used to detect mutations, while dbSNP (http://www.ncbi.nlm.nih.gov/projects/SNP) was used as a reference for recorded SNPs. Data from the 1000 Genomes (http://www.1000genomes.org), HapMap (ftp://ftp.ncbi.nlm.nih.gov/hapmap) and BGI’s in-house databases were used to investigate the possible pathogenicity of the variants detected in this sequencing approach. The American College of Medical Genetics and Genomics Guideline was used as the reference for data interpretation.

Our results revealed that the proband carried compound heterozygous mutations in the *MYO15A* gene (Fig. 1e). Occurring in exon 33, the c.6892C > T mutation, which was inherited from the unaffected mother (I-2), led to a premature stop codon at amino acid position 2298 for the MYO15A protein. The c.10251_10253delCTT mutation, which was located in exon 63 and passed on from his clinically normal father (I-1), led to a deletion of phenylalanine at position 3420. To verify mutations in the *MYO15A* gene (NCBI Reference Sequence: NM_016239.3), PCR primers were designed by Primer3 (http://primer3.ut.ee/) online software and used for amplification. Forward: 5′-TCCCTCATTTCCATTCCTGTG-3′ and Reversed: 5′-CCATTTGTACCGTCCTGATTG-3′ for the c.6892C > T mutation; Forward: 5′-CTGCCTGGAGAAAACATGTCTT-3′ and Reversed: 5′- AAAGAAACCAAACCTGCTGACA-3′ for the c.10251_10253delCTT mutation. Sanger sequencing confirmed that these two *MYO15A* gene variants co-segregated with deafness in this family. Both mutations were absent in 200 ancestry-matched, unrelated subjects. Further supporting our findings, is the fact that the dizygotic twins (II-2 and II-3), both of which were heterozygous carriers, each passed the newborn hearing screening tests and had normal hearing.

### Bioinformatics

**Fig. 2:**
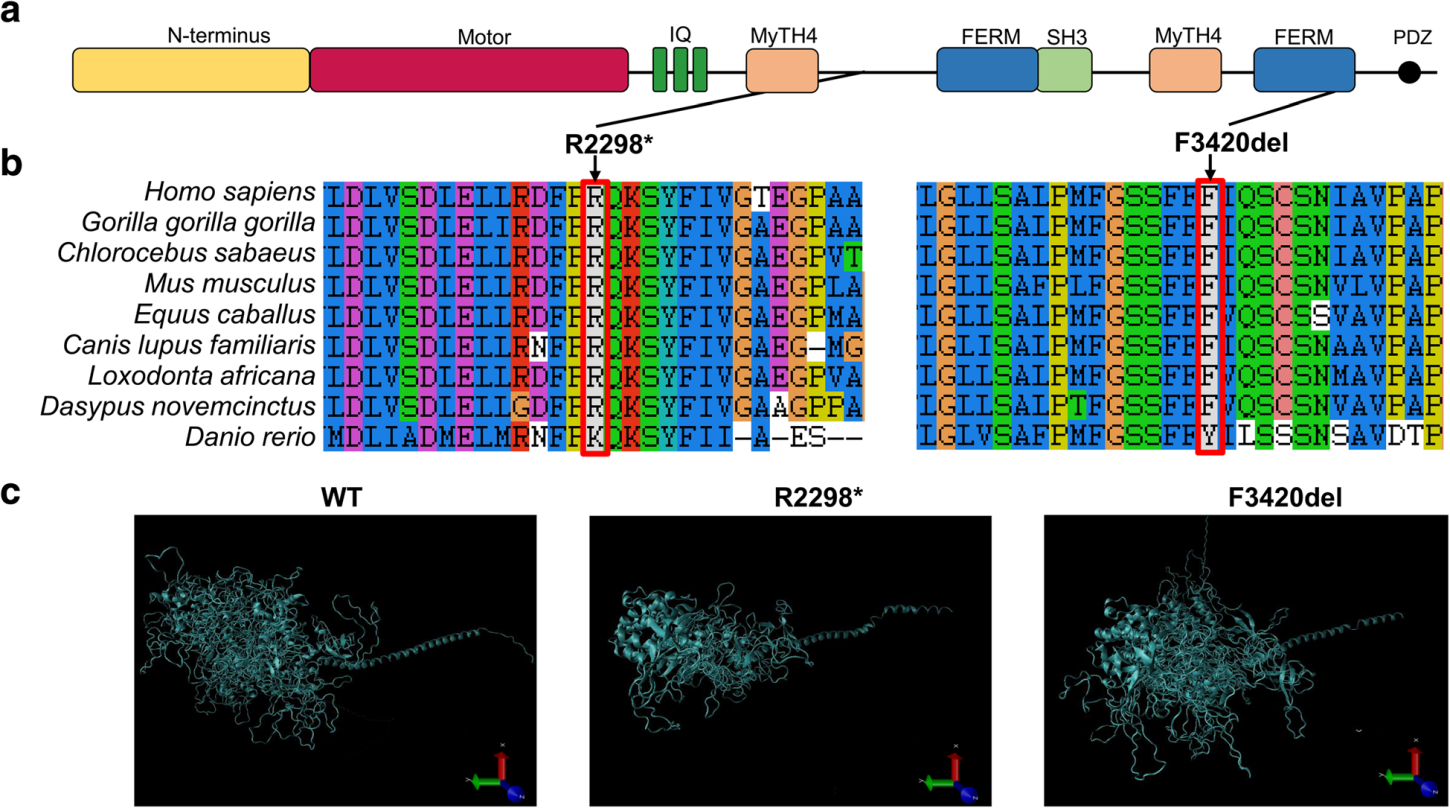
Functional analysis of the MYO15A mutant protein. **a** Schematic representation of MYO15A protein domain structure. A motor domain, three IQ motifs, two MyTH4 domains, two FERM domains, a SH3 domain, and a PDZ domain are depicted. **b** Multiple sequence alignment showed that R2298 and p.F3420del are positioned in a highly conserved region. Changes in amino acids are highlighted in the red boxes. **c** Molecular modeling revealed that the p.R2298* and p.F3420del mutants affect the normal protein structure of MYO15A

The MYO15A protein contains an N-terminal, a motor domain, three light-chain binding motifs (IQ), two myosin-tail homology 4 (MyTH4) domains, two band 4.1, ezrin, radixin, moesin (FERM) domains, a Src-homology-3 (SH3) domain, and a C-terminal PDZ ligand motif (Fig. 2a). The multiple MYO15A amino acid sequences alignment revealed that the arginine at position 2298 and the phenylalanine at position 3420 are highly conserved among mammalian species (Fig. 2b). The prediction of the effects that candidate mutations may have were evaluated with SIFT (http://sift.bii.a-star.edu.sg/) and Mutation Taster (http://www.mutationtaster.org/) programs. Using Mutation Taster, the c.6892C > T and c.10251_10253delCTT variants were predicted to be disease causing with scores of 1 and 0.999, respectively. Moreover, SIFT indicated that c.10251_10253delCTT was damaging for the *MYO15A* function.

The templates for the three-dimensional structures of the wild type, p.R2298* mutant, and p.F3420del mutant MYO15A proteins were searched for by using SWISS MODEL (http://swissmodel.expasy.org). Myosin-VA (pdb ID: 2dfs.1.A), myosin-VIIa isoform 1 (pdb ID: 3pvl.1.A) and the unconventional myosin-VIIa (pdb ID: 5mv9.1.A) structures were selected because templates with high sequence identity covering the amino acid positions of our variants were not available. The wild type and mutant models were built via the MODELLER software package. Compared with the three-dimensional structure of the wild-type MYO15A protein, the p.R2298* mutant protein structure was incomplete and the p.F3420del mutant protein structure was dramatically changed (Fig. 2c).

## Discussion and conclusions

Hereditary non-syndromic hearing loss is highly heterogeneous. By the time we provided genetic counseling, the mother of the proband was pregnant. The conventional genetic strategy for screening the causative mutations in this ARNSHL family is time-consuming and impractical. Therefore, we conducted target capture sequencing, which is more suitable for clinical diagnosis with higher sequencing depth and lower costs over whole-exome sequencing.

In this case, we identified compound heterozygous mutations in the *MYO15A* gene of the proband. These mutations included a novel nonsense mutation, c.6892C > T reported here for the first time and another potentially pathogenic deletion, c.10251_10253delCTT, previously described by Yang et al. [12]. The ASSR audiogram showed severe hearing loss in the proband. Moreover, his ABR and DPOAE tests were negative despite high levels of stimulation, indicating profound deafness, which is consistent with the *MYO15A*-related prelingual deafness phenotypes.

Myosin belongs to the actin-based motor molecule superfamily and plays an important role in normal auditory function. Unconventional myosins serve in intracellular movements since their highly divergent tails bind to membranous compartments [13]. Mutations in five unconventional myosin genes *MYOIA*, *MYOIIIA*, *MYOVI*, *MYOVIIA*, and *MYOXVA* have been reported to cause hearing loss.

*MYO15A* is located at 17p11.2, which has 66 exons encoding for 3530 amino acids. This protein is expressed at the tips of inner ear sensory cells. The tip is the site of stereocilia growth and is necessary for actin microfilament organization [14]. Shaker-2 mice harboring *MYO15A* mutations [15], exhibited profound deafness and had much shorter stereocilia than wild-type mice [16]. Thus, the myosin XVa protein is critical for the elongation and differentiation of inner ear hair cells and is required for the arrangement of stereocilia in mature hair bundles [17].The MYO15A protein consists of multiple domains, which include an N-terminal and a motor domain, a neck region and a tail region [18]. Pathogenic mutations, which have no preferential distribution within the *MYO15A* gene, have been found in all domains [19].

The p.R2298* mutation was observed between the first MyTH4 and FERM domains. The premature termination of translation resulted in the loss of the tail region of the MYO15A protein and the consequential loss of normal protein function. The SH3-MYTH4-FERM tail region domain is also known for its ability to bind to whirlin [20], which is necessary for the elongation and maintenance of hair cell stereocilia in the organ of Corti. Truncated proteins often lead to nonsense-mediated decay and the consequential loss of normal protein function. Several other nonsense mutations of *MYO15A* gene, including p.W1975∗, p.S2661*, p.R2923*, p.Y2819* and p.Q3264* [19, 21, 22], have been reported to cause hearing loss. The damaged domain caused by p.R2298* mutation is larger than those by many other known pathogenic nonsense variants, leading one to conclude that the nonsense mutation reported here would definitely lead to a pathogenic state. The second mutation p.F3420del was reported previously in an epidemiology study. It is located in the second FERM domain of the MYO15A protein. Previous reports suggested that the MyTH4-FERM domain is required for its localization to stereocilia tips and enriching the adhesion molecules, actin-regulatory proteins [23]. Mutations in this domain could also r result in losing the normal function of the MYO15A protein. In addition, bioinformatics analysis using Mutation Taster and SIFT supported the hypothesis of pathogenicity of both variants. Structural modeling demonstrated that the p.R2298* and p.F3420del variants altered the normal MYO15A protein structure.

Collectively, our study demonstrated that the compound heterozygous c.6892C > T and c.10251_10253delCTT mutations in *MYO15A* gene were the pathogenic variants in this ARNSHL family. The c.6892C > T nonsense mutation is reported for the first time. Data presented here extend the pathogenic mutation spectrum of the *MYO15A* gene, which has implications in genetic counseling for hereditary deafness. Functional investigations are needed to further elucidate the pathogenic mechanism of these two mutations.

## Additional files

Additional file 1: **Table S1.** Summary of 127 hereditary deafness-related genes by target region capture sequencing. (DOCX 16 kb) (DOCX 15 kb)

Additional file 2: **Table S2.** Overview of target region capture sequencing results of the proband affected by ARNSHL. (DOCX 15 kb) (DOCX 14 kb)

## Abbreviations

ABR: Auditory brainstem responses
ARNSHL: Autosomal recessive non-syndromic hearing loss
ASSR: Auditory steady state response
CT: Computed tomography;
DFNB: Autosomal recessive non-syndromic hearing loss
DPOAE: Distortion product otoacoustic emission
FERM: Band 4.1, ezrin, radixin, moesin domain
MyTH4: Myosin-tail homology 4 domain
SH3: Src-homology-3 domain

## References

[1] Fortnum H, Summerfield A, Marshall D, Davis A, Bamford J (2001). Prevalence of permanent childhood hearing impairment in the United Kingdom and implications for universal neonatal hearing screening: questionnaire based ascertainment study. BMJ.

[2] Morton C, Nance W (2006). Newborn hearing screening--a silent revolution. N Engl J Med.

[3] Nance W, Lim B, Dodson K (2006). Importance of congenital cytomegalovirus infections as a cause for pre-lingual hearing loss. J Clin Virol.

[4] Shearer A, Smith R (2012). Genetics: advances in genetic testing for deafness. Curr Opin Pediatr.

[5] Duman D, Tekin M (2012). Autosomal recessive nonsyndromic deafness genes: a review. Front Biosci (Landmark Ed).

[6] Hilgert N, Smith RJ, Van Camp G (2009). Forty-six genes causing nonsyndromic hearing impairment: which ones should be analyzed in DNA diagnostics?. Mutat Res.

[7] Friedman T, Liang Y, Weber J, Hinnant J, Barber T, Winata S, Arhya I, Asher J (1995). A gene for congenital, recessive deafness DFNB3 maps to the pericentromeric region of chromosome 17. Nat Genet.

[8] Wang A, Liang Y, Fridell R, Probst F, Wilcox E, Touchman J, Morton C, Morell R, Noben-Trauth K, Camper S (1998). Association of unconventional myosin MYO15 mutations with human nonsyndromic deafness DFNB3. Science.

[9] Winata S, Arhya I, Moeljopawiro S, Hinnant J, Liang Y, Friedman T, Asher J (1995). Congenital non-syndromal autosomal recessive deafness in Bengkala, an isolated Balinese village. J Med Genet.

[10] Atik T, Bademci G, Diaz-Horta O, Blanton S, Tekin M (2015). Whole-exome sequencing and its impact in hereditary hearing loss. Genet Res (Camb).

[11] Miyagawa M, Naito T, Nishio SY, Kamatani N, Usami S (2013). Targeted exon sequencing successfully discovers rare causative genes and clarifies the molecular epidemiology of Japanese deafness patients. PLoS One.

[12] Yang T, Wei X, Chai Y, Li L, Wu H (2013). Genetic etiology study of the non-syndromic deafness in Chinese Hans by targeted next-generation sequencing. Orphanet J Rare Dis.

[13] Li J, He Y, Weck M, Lu Q, Tyska M, Zhang M (2017). Structure of Myo7b/USH1C complex suggests a general PDZ domain binding mode by MyTH4-FERM myosins. Proc Natl Acad Sci U S A.

[14] Belyantseva IA, Boger ET, Naz S, Frolenkov GI, Sellers JR, Ahmed ZM, Griffith AJ, Friedman TB (2005). Myosin-XVa is required for tip localization of whirlin and differential elongation of hair-cell stereocilia. Nat Cell Biol.

[15] Liang Y, Wang A, Probst FJ, Arhya IN, Barber TD, Chen KS, Deshmukh D, Dolan DF, Hinnant JT, Carter LE (1998). Genetic mapping refines DFNB3 to 17p11.2, suggests multiple alleles of DFNB3, and supports homology to the mouse model shaker-2. Am J Hum Genet.

[16] Probst F, Fridell R, Raphael Y, Saunders T, Wang A, Liang Y, Morell R, Touchman J, Lyons R, Noben-Trauth K (1998). Correction of deafness in shaker-2 mice by an unconventional myosin in a BAC transgene. Science.

[17] Belyantseva IA, Boger ET, Friedman TB (2003). Myosin XVa localizes to the tips of inner ear sensory cell stereocilia and is essential for staircase formation of the hair bundle. Proc Natl Acad Sci U S A.

[18] Karolyi IJ, Probst FJ, Beyer L, Odeh H, Dootz G, Cha KB, Martin DM, Avraham KB, Kohrman D, Dolan DF (2003). Myo15 function is distinct from Myo6, Myo7a and pirouette genes in development of cochlear stereocilia. Hum Mol Genet.

[19] Rehman AU, Bird JE, Faridi R, Shahzad M, Shah S, Lee K, Khan SN, Imtiaz A, Ahmed ZM, Riazuddin S (2016). Mutational Spectrum of MYO15A and the molecular mechanisms of DFNB3 human deafness. Hum Mutat.

[20] Delprat B, Michel V, Goodyear R, Yamasaki Y, Michalski N, El-Amraoui A, Perfettini I, Legrain P, Richardson G, Hardelin JP (2005). Myosin XVa and whirlin, two deafness gene products required for hair bundle growth, are located at the stereocilia tips and interact directly. Hum Mol Genet.

[21] Fattahi Z, Shearer AE, Babanejad M, Bazazzadegan N, Almadani SN, Nikzat N, Jalalvand K, Arzhangi S, Esteghamat F, Abtahi R (2012). Screening for MYO15A gene mutations in autosomal recessive nonsyndromic, GJB2 negative Iranian deaf population. Am J Med Genet A.

[22] Chang MY, Lee C, Han JH, Kim MY, Park HR, Kim N, Park WY, Oh DY, Choi BY (2018). Expansion of phenotypic spectrum of MYO15A pathogenic variants to include postlingual onset of progressive partial deafness. BMC medical genetics.

[23] Weck M, Grega-Larson N, Tyska M (2017). MyTH4-FERM myosins in the assembly and maintenance of actin-based protrusions. Curr Opin Cell Biol.

