# Supplementary material for: A novel nonsense mutation in MYO15A is associated with non-syndromic hearing loss: a case report
Source: BMC Med Genet. 2018 Aug 1;19:133. doi: 10.1186/s12881-018-0657-y (PMC6090657; doi:10.1186/s12881-018-0657-y)
Supplement: Supplementary file 1 — Table S1. Summary of 127 hereditary deafness-related genes by target region capture sequencing. (DOCX 16 kb) (DOCX 15 kb) [file 12881_2018_657_MOESM1_ESM.docx]

**A novel nonsense mutation in *MYO15A* is associated with non-syndromic hearing loss**

Di Ma, Shan-Shan Shen, Hui Gao, Hui Guo, Yu-Mei Lin, Yu-Hua Hu, Ruan-Zhang Zhang, Sha-Yan Wang*

Shenzhen People’s Hospital, Clinical Medical College of Jinan University, Dongmen North Rd. 1017, Shenzhen 518020, PR China

* Corresponding author. Fax: +86 755 25633958.

E-mail address: shayanw@163.com (S.-Y. Wang).

**Table S1.Summary of 127 deafness-related genes by target capture sequencing**

| Name | Genes |
| --- | --- |
| Autosomal recessive nonsyndromic hearing impairment | *GJB2,GJB6,MYO7A,MYO15A,FOXI1,KCNJ10,SLC26A4,TMIE,TMC1,TMPRSS3,OTOF,CDH23,ATP2B2,GIPC3, STRC,USH1C,OTOG,TECTA,OTOA,PCDH15,RDX,GRXCR1,TRIOBP,CLDN14,MYO3A,DFNB31,ESRRB,ESPN,MYO6,HGF,GJA1,ILDR1,MARVELD2,DFNB59,SLC26A5,LRTOMT,LHFPL5,BSND,MSRB3,LOXHD1,TPRN,GPSM2,PTPRQ, SERPINB6,GJB3* |
| Autosomal dominant nonsyndromic hearing impairment | *ACTG1,CCDC50,CEACAM16,COCH,CRYM,DFNA5,D1ABLO,D1APH1,DSPP,EYA4,GJB2,GJB3,GJB6,GRHL2,KCNQ4,MIR96,MYH14,MYH9,MYO1A,MYO6,MYO7A,POU4F3,SIX1,SLC17A8,TECTA,TJP2,TMC1,WFS1,D1APH3* |
| X-link hereditary hearing impairment | *POU3F4,SMPX,PRPS1* |
| Maternally inherited hearing impairment | [*MTRNR1*](http://www.ncbi.nlm.nih.gov/omim/561000)*,MTTS1* |
| Syndromic hearing impairment | *SERAC1,PDSS1,FGFR3,FGFR2,FGFR1,PHEX,DLX5,TNFRSF11B,COL2A1,COL11A1,COL9A1,COL9A2,COL4A3,COL4A4,COL4A5,BSND, PAX2,SOX9,GATA3,SLC19A2,IGF1,PAX3,MITF,SNAI2,EDNRB,EDN3,SOX10,HOXA1,SOBP,EYA1,SIX5,SIX1,CHD7, SEMA3E, SMAD4, FGF3,TCOF1,PRRX1,GLI3,HOXA2,KCNE1,KCNQ1,CACNA1D,ALMS1, LRP2,TIM8A, NDP, WFS1, OPA1, SLC4A11, MYO7A, USH1C, CDH23,PCDH15,USH1G,USH2A,GPR98,PDZD7,DFNB31,CLRN1,MTTE,MTTL1,MTTK,SLC26A4,KCNJ10,FOXI1* |
