# Supplementary material for: A novel nonsense mutation in MYO15A is associated with non-syndromic hearing loss: a case report
Source: BMC Med Genet. 2018 Aug 1;19:133. doi: 10.1186/s12881-018-0657-y (PMC6090657; doi:10.1186/s12881-018-0657-y)
Supplement: Supplementary file 2 — Table S2. Overview of target region capture sequencing results of the proband affected by ARNSHL. (DOCX 15 kb) (DOCX 14 kb) [file 12881_2018_657_MOESM2_ESM.docx]

**A novel nonsense mutation in *MYO15A* is associated with non-syndromic hearing loss**

Di Ma, Shan-Shan Shen, Hui Gao, Hui Guo, Yu-Mei Lin, Yu-Hua Hu, Ruan-Zhang Zhang, Sha-Yan Wang*

Shenzhen People’s Hospital, Clinical Medical College of Jinan University, Dongmen North Rd. 1017, Shenzhen 518020, PR China

* Corresponding author. Fax: +86 755 25633958.

E-mail address: shayanw@163.com (S.-Y. Wang).

**Table S2.Target capture sequencing results of the proband affected by ARNSHL**

| **Parameter** | **II-1** |
| --- | --- |
| Number of target genes | 127 |
| Target region yield (bp) | 619167 |
| Coverage of target region | 98.33% |
| Mean read depth of targeted region | 349.88 |
| Mean read depth > 30X | 96.75% |
